# An Outlook on the Complexity of Protein Morphogenesis in Health and Disease

**DOI:** 10.3389/fmolb.2022.909567

**Published:** 2022-06-13

**Authors:** Maurizio Brunori, Stefano Gianni

**Affiliations:** ^1^ Istituto Pasteur-Fondazione Cenci Bolognetti, Dipartimento di Scienze Biochimiche “A. Rossi Fanelli” and Istituto di Biologia e Patologia Molecolari del CNR, Sapienza Università, Rome, Italy; ^2^ Accademia Nazionale dei Lincei, Rome, Italy

**Keywords:** folding, aggregation, stability, misfolding, retromer

## Abstract

The study of the mechanisms whereby proteins achieve their native functionally competent conformation has been a key issue in molecular biosciences over the last 6 decades. Nevertheless, there are several debated issues and open problems concerning some aspects of this fundamental problem. By considering the emerging complexity of the so-called “native state,” we attempt hereby to propose a personal account on some of the key topics in the field, ranging from the relationships between misfolding and diseases to the significance of protein disorder. Finally, we briefly describe the recent and exciting advances in predicting protein structures from their amino acid sequence.

## Introduction

One of the most fascinating topics in life sciences lies in the understanding of the processes of self-assembly of supramolecular architectures. Because of its vital importance in essentially all cellular pathways, the study of the folding of proteins has undoubtedly taken center stage in biomolecular sciences over the last few decades (Eaton 2021). The birth date of the protein-folding problem has been assigned to the publication of the pioneering article by Anfinsen, Haber, Sela, and White on the refolding of denatured ribonuclease on 15 September 1961 (Anfinsen et al., 1961), which therefore last year celebrated its 60th anniversary. In 1972, half a century ago, Chris Anfinsen was awarded the Nobel prize in Chemistry with the following motivation: *for his work on ribonuclease, especially concerning the connection between the amino acid sequence and the biologically active conformation.*

There were two critical breakthroughs arising from this work: first, it was unambiguously demonstrated that a protein might refold spontaneously to its physiologically active state, a statement that, incidentally, fits the concept of *substance* as defined by Aristotle (*Substantia causa sui*); second, by analyzing the dependence of the enzymatic activity versus the time of reoxidation, the authors could contribute the first attempt to describe the order of events of the molecular pathway leading to the biologically active state. Noteworthy, these aspects reflect the bi-faceted nature of the protein-folding problem—the description of i) the amino acid code that specifies for a given structure and ii) the mechanism by which this state is achieved.

In a nutshell, protein folding is the reaction leading a polypeptide from disorder to order, from unfolded to folded. In his seminal assay *“Case and Necessity*,*”* Jacques Monod suggested that one of the key foundations of life is in the ability to spontaneously reach an active state (self-organization of its constituents). Thus, folding is the manifestation of the *morphogenesis of a protein*, whereby an amino acid sequence is able to self-assemble into a so-called *native conformation*, from Latin “nativus—born,” which is the biologically active state endowed of a specific function. Whilst the native conformation of a protein is generally assigned to its precise three-dimensional structure as obtained, for example, by X-ray diffraction in a crystal, intense research carried out over the last few decades has tremendously increased our understanding of its complexity. In this mini-review, we attempt to offer a critical outlook on some of the key aspects arising from the study of such complexity, *vis-a-vis* the essence of the protein-folding problem, as framed by the pioneering work of Anfinsen and co-workers. Furthermore, we attempt to highlight some key questions that, in our opinion, demand to be elucidated.

### Native State in Solution

Since life primarily occurs in hydration, the maintenance of protein solubility is an essential feature to achieve the native condition. It is generally known that native globular proteins are highly soluble in water. For example, in the human red blood cell, hemoglobin A is at a concentration of >100 g L^−1^, not far from solubility (Antonini and Brunori 1971). Intracellular precipitation of the protein is avoided because of (i) the simultaneous presence of oxy and deoxy hemoglobins that have different solubility (Adachi and Asakura 1979) and (ii) the continuous mixing of the intracellular components due to squeezing of the erythrocytes while traveling through the capillaries and more. In other species, intracellular precipitation is contrasted by the presence of more than one hemoglobin component having similar functional properties but different amino acid sequences.

The great solubility in water of native globular proteins may appear in contrast with the statement made long ago by Linus Pauling that: *“… proteins are sticky*.*”* It is common practice that heating leads to coagulation and massive aggregation of unfolded, denatured proteins, the condensed state being stabilized by-and-large by hydrophobic interactions. Biochemists have often been discouraged, if not irritated, by the unwanted precipitation of an interesting protein that may occur during purification; this is why a cold room is classically present in every biochemistry institute.

Of interest, in the cellular environment, it has been reported that the expression of human genes is anti-correlated with aggregation propensity of their phenotypes (Tartaglia et al., 2007; Vecchi et al., 2020). This finding indicates that human proteins evolved to escape the formation of aggregates. Accordingly, detection of supersaturated proteins in specific tissues may represent a possible source of vulnerability and could reveal a potential link to cellular dysfunction (Ciryam et al., 2016; Kundra et al., 2017; Yerbury et al., 2019). Additionally, it was observed that minor changes to the amino acid composition on the surface of proteins can dramatically change their solubilities (Garcia-Seisdedos et al., 2017; Garcia Seisdedos et al., 2022), illustrating the fine balance of forces driving protein aggregation. Thus, as briefly recapitulated as follows, it is of great importance to understand the complex scenario that links the thermodynamics of the soluble native state to the formation of aberrant insoluble structures.

### Protein Aggregation: The Amyloid State and Cell Toxicity

**FIGURE 1:**
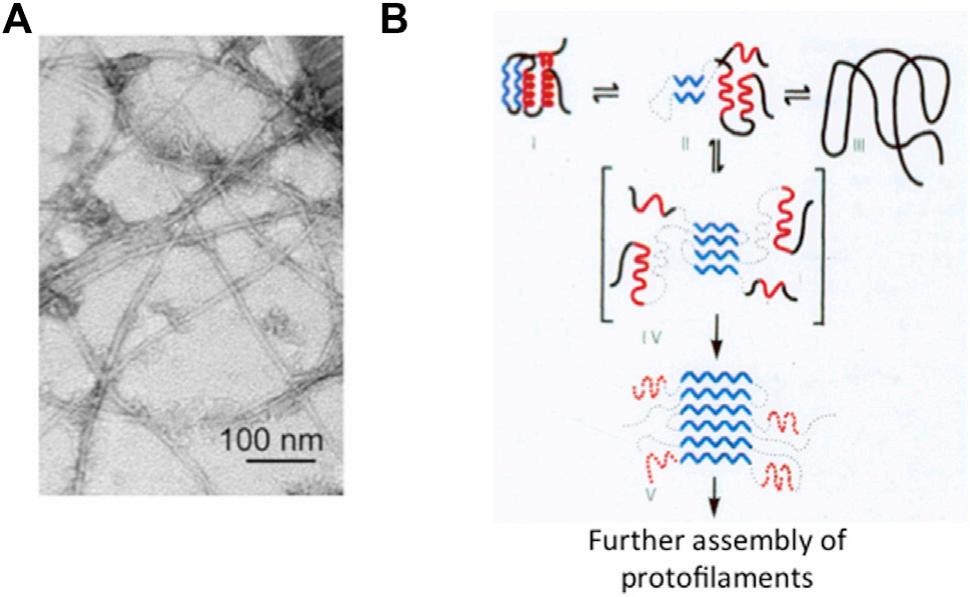
Protein aggregation and misfolding. Panel **(A)** Electron microscopy image of the amyloid state of Aβ, adapted from Lu et al. (2013). As discussed in the text, it appears that in spite of differences in the amino acid sequences or in the structure of the protein involved, the typical amyloid state consists of thread-like fibrils of about 10 nm diameter and rich in β-sheet structure. Panel **(B)** Schematic representation of the folding of a small globular protein and the formation of amyloid. The native state (depicted on the top left) consists of two α-helices (in red) and two β-strands (in blue). Aggregation and formation of the amyloid is triggered by the accumulation of a misfolded intermediate (in square parenthesis).

The end of the last century has witnessed a neat revolution in our understanding of native state thermodynamics and stability. In fact, whilst Anfinsen’s experiment would theoretically imply that the native state is the most stable conformation of a given polypeptide chain, it was observed that a protein may form fibrillar aggregates displaying significantly higher stabilities (Prusiner 1985; Canet et al., 1999; Chiti and Dobson 2006; Tycko and Wyckner 2013). This type of state, built by a single specific protein polymerizing into (intra or inter) cellular long filaments with a characteristic 3D structure, has been called an *amyloid*, a generic name adopted after Rudolf Virkow in 1853 (Figure 1).

The association between amyloid and human disease dates back to the early identification by Dr. Alois Alzheimer of mysterious aggregates in the brain of Mme. Auguste Deter, who died in her fifties with a devastating dementia, nowadays carrying the name of the discoverer. However, an increasing number of globular proteins with no obvious connection to pathology has been shown to have an intrinsic propensity to yield, under appropriate conditions, fibrillar aggregates with the characteristic overall morphology of an amyloid (Chiti et al., 2002; Chiti and Dobson 2009; Knowles et al., 2014; Dobson et al., 2020). This suggested that the inclination to form an amyloid is an inherent generic property of virtually all proteins, although the propensity to aggregate can vary dramatically with sequences and conditions. Chris Dobson summarized the matter by stating that *“… in principle every protein can assemble into an amyloid state*.*”*

From a structural perspective, an amyloid filament is generally contributed by a single type of protein assembled in a thread-like structure of approximately 7–13 nm diameter, formed by protofilaments associated laterally (Balbach et al., 2000; Eisenberg and Juncker 2012; Tycko and Wyckner 2013; Iadanza et al., 2018; Gallardo et al., 2020). The stability of the fibrils depends on hydrophobic interactions and H-bonds along the main axis. Association of protofilaments leads to an amyloid whose thermodynamic stability exceeds that of the originating native protein. In addition, the barrier to depolymerization is very high making the reaction essentially irreversible (Chiti and Dobson 2009).

The monotony of the basic architecture led to believe that in all cases the 3D structure is essentially the same (Brunori 2021). However studies by solid state NMR and cryo-electron microscopy have indicated that despite recurrent canonical features in the morphology, the 3D structure displays a certain degree of polymorphism for different types of amyloid (*ex vivo or in vitro;* from the brain of cadavers or assembled *in vitro* from synthetic polypeptides) (Colvin et al., 2016; Wälti et al., 2016; Cao et al., 2021; Iakovleva et al., 2021; Yang et al., 2022)). This is particularly evident when an amyloid is assembled from shorter peptides, but it is also seen with whole proteins such as Tau or α-synuclein (Ulamec et al., 2020). In some cases, polymorphism has been correlated to the binding of different cofactors or truncation of a domain or interactions with cellular components (Gallardo et al., 2020). Notably, it is still unknown whether the amyloid made of a single protein may or may not display differences in the onset of cellular dysfunction or in the progression of a disease, which may be correlated to polymorphism.

Many biochemists have been attracted over the years by the significance of amyloids in the brains of people those died of one or another disease associated to neurodegeneration and eventually dementia. This group of pathologies expanded with time, from the more frequent syndromes named after Dr. Alzheimer and Dr. Parkinson to many others affecting not only the central or peripheral nervous tissue(s) but also other organs in the body such as the pancreas, the heart, and more (Kelly 1998; Chiti and Dobson 2006; Chiti and Dobson 2009; Singh et al., 2021; Cascella et al., 2022; Chiti and Kelly 2022; Koopman et al., 2022). At the very beginning of this century, there was enough reliable information to pursue and defend a general view assigning a central role to (i) the aggregation of a specific protein typical of a specific neurodegenerative disease; (ii) the correlation between the intrinsic propensity of a protein to aggregate and single site mutations that further reduce solubility; and (iii) the population of misfolded states (often quite compact) in the mechanism of formation of the kernel and the extended amyloid, largely by β-sheet interactions. In fact, this class of devastating fatal diseases which prevail in older people (see below) are nowadays called *misfolding disorders* (Chiti and Dobson 2006; Knowles et al., 2014; Chiti and Kelly 2022). In summary, although aggregation may initiate starting from different states (native, denatured, or intermediate), clear-cut data indicate that even elusive misfolded intermediates play a significant role in the formation of amyloids (Figure 1) (Kelly 1998; Chiti and Dobson 2006; Chiti and Dobson 2009; Knowles et al., 2014; Dobson et al., 2020; Cawood et al., 2021).

A quantitative description of the complex aggregation kinetics is very difficult, and a dissection of the microscopic events involved is very challenging (Buell et al., 2014). The overall time course seems to mimic the one reported for the polymerization of the deoxygenated sickle cell hemoglobin single mutant ((Hofrichter et al., 1974; Eaton and Hofrichter 1990). The formation of the amyloid follows a time lag whose length depends on the specific protein involved and on its concentration. It is accepted that a classical nucleation–propagation mechanism is inadequate to fit experimental data, and a crucial role for filament fragmentation and secondary nucleation has been advocated. Determination of the rate constants for the microscopic steps and their dependence on experimental conditions is very difficult; the nonlinear nature of the rate equations makes it difficult to apply a rigorous quantitative analysis. Breaking down the significant parameters demands (a) extensive experiments to unveil the role of protein concentration and seeding on the time course of aggregation and (b) very sophisticated mathematical analysis.

Unveiling the mechanism and sorting out intermediates is crucial to combat the disease because of the following reasons: the first goal is to search for drugs or antibodies that may bind with high affinity to the misfolded monomers or oligomers and thereby interfere with polymerization; clearly, this demands the identification and structural characterization of reaction intermediates and their kinetics of formation and interconversion. The search to discover molecules to combat neurodegeneration following this strategy seems to produce some positive results. Dobson and co-workers provided self-consistent solutions of the equations describing the steps of primary and secondary nucleation, as well as fragmentation of the growing fibers (Knowles et al., 2009). By systematically dissecting each microscopic step in the mechanism of aggregation of Aβ42 (the peptide forming the amyloid in Alzheimer’s disease), it was discovered that bexarotene, an approved anticancer drug, may act as a controller of the primary nucleation, thus delaying the formation of larger aggregates and toxic species (Habchi et al., 2016; Ruggeri et al., 2021).

An important second goal is to tackle the problem of identifying the toxic species and defining the mechanism of cellular toxicity. The hypothesis that brain damage was caused primarily, if not exclusively, by the extensive brain plaques or intracellular fibrillary tangles has lost some of the initial relevance. Recent data suggest that some of the polymerization intermediates are likely to be the main species toxic to neurons (Fusco et al., 2017; Cascella et al., 2019; Venkatramani et al., 2022); in particular, the oligomers are presumed to increase the level of cellular damage because of their propensity to bind to and diffuse inside the cellular membrane and kill the cell. Moreover, they are crucial to the multiplication of the amyloid being able to condense into new kernels starting the formation of secondary protofibrils.

### Why Is Aging Associated With an Increase in Misfolding Diseases?

Neurodegenerative diseases are quite frequent in older people, with a devastating impact on individuals, families, and societies. It has been estimated that Alzheimer’s disease alone will affect between one-third and one-half of people above 85 years of age. Currently, these diseases have *no cure*; therefore, the individual, social, and financial burden of assisting these disabled patients will grow, and by 2050, the economic toll is expected to rise to about one trillion US$ per year in the USA alone. In spite of obvious clinical differences—with symptoms ranging from progressive dysfunction of motor control to mood disorders and cognitive deficits, and eventually full-blown dementia—at the molecular and cellular levels, these disabling diseases display some fundamental commonalities (Small and Petsko 2015; Brunori 2021). As outlined previously, an important step forward has been the discovery that protein misfolding is likely to be a unifying molecular mechanism. Unveiling the molecular, genetic, and cellular commonalities of these age-related disorders and discovering methods and drugs that either prevent or interfere with the formation and accumulation of misfolded proteins are mandatory.

The mechanism whereby longevity affects the onset of a disease is still somewhat of a conundrum. The likelihood to produce intracellular or intercellular amyloids depends to a first approximation on a few factors, starting with the intrinsic propensity to aggregate of the protein/peptide involved. Quite often, a mutation of the protein in question is associated to a considerable increase in the propensity to aggregate and clinically with an early onset of the disease. This effect, discovered long ago for synuclein which forms the Levy’s bodies typical of Parkinson’s disorder (Goedert et al., 2017), has been observed for most other cases including, for example, the *amyloid precursor protein* (APP) involved in Alzheimer’s and *superoxide dismutase* (SOD) involved in a quota of amyotrophic lateral sclerosis *(*ALS) (Morrison and Morrison 1999; Nordlund and Oliveberg 2006). It should not be overlooked that a mutant protein endowed with an increased propensity to aggregate is synthesized from the time of conception, yet deposition of the fibrils almost always begins late in life—why?

At present, the aging-dependent cellular processes that jeopardize the efficiency of the homeostasis network are not fully clear (Brunori 2021). A major task is to unveil the biochemical mechanisms responsible for the enhanced rate of formation of the amyloidogenic proteins/peptides or the decrease in efficiency of clearance of the misfolded states. Protein homeostasis is a complex biochemical network whose job is cleaning the cell from molecular garbage. Under normal conditions, the potentially amyloidogenic proteins/peptides are processed *via* the physiological degradation paths. With aging however, proteostasis may begin to decline with an increase in the concentration of the amyloidogenic species and formation of kernels which promote polymerization and subsequently fragmentation, with an increase in the oligomeric toxic species.

Aging may depend on deterioration of several different components of the homeostasis network and a decrease of the necessary biological energy supply. The complexity of neurodegenerative disorders that deserves the highest priority along two main lines: (i) to understand the biochemical mechanisms responsible for the decline in the clearance efficiency of the amyloidogenic events and to device a protocol to rescue control; (ii) to unveil the reactions responsible for the increase in the rate of production of the amyloidogenic species in an attempt to interfere with accumulation of toxic oligomers, thereby reducing the rate of fibrillation.

**FIGURE 2:**
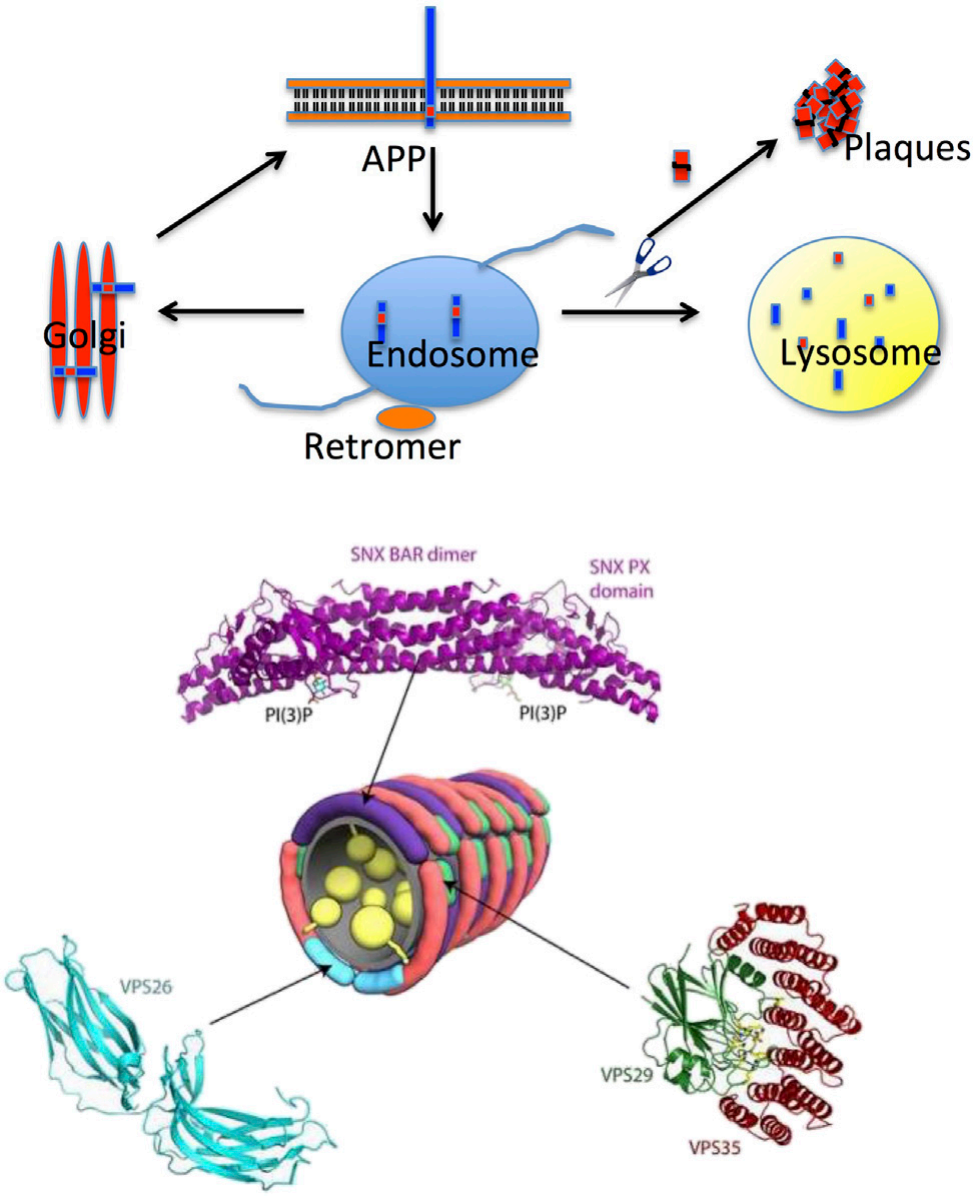
Intracellular protein trafficking and the retromer. *Top panel*: the retromer is part of the endosome, the hub in intracellular protein trafficking; retromer’s role is to transfer proteins to the Golgi apparatus or to the lysosome for proteolysis. Retromer’s dysfunction in Alzheimer’s disease causes improper sorting of APP (the amyloid precursor protein) with accumulation of Aβ40/42 and eventually plaque formation (inspired by a scheme suggested by Prof. G Petsko, New York, USA, reproduced with permission). *Bottom panel*: structural organization of the retromer. The crystal structure of human VPS6A is shown in cyan (PDB 2FAU), and those of human VPS29 and VPS35 are shown in green and red, respectively (PDB 2R17). Residues surrounding the inactive metallophosphoesterase site of VP29 are shown in a stick model. The structure of the SNX dimer is represented in purple (Adapted from Brunori and Gianni (2016); reported with permission).

An interesting hypothesis that emerged at the end of the last century is based on the age-dependent dysfunction of the retromer (Seaman et al., 1998; Seaman 2012), a complex protein assembly which is crucial for endosomal protein trafficking, including the intracellular sorting and recycling of the APP (Figure 2). Early on, it was observed by optical microscopy of the Alzheimer’s brain that endosomes, the intracellular hub collecting membrane proteins to be recycled and physiologically addressed to the Golgi, appeared damaged. More recently, it was shown by genetic and functional studies that retromer dysfunction is linked to Alzheimer’s disease and (afterward) to Parkinson’s disease as well (Small and Petsko 2015; Small et al., 2017; Small and Petsko 2020). In the last few years, the number of neurological disorders linked to a retromer dysfunction has increased considerably, including Down syndrome (Curtis et al., 2022). From a structural perspective, the retromer complex appears to be assembled in an arch-shaped scaffold with highly dynamic properties (Chandra et al., 2020). Furthermore, it appears that the retromer exists as monomers and low-order oligomers (dimers, trimers, and tetramers) when interacting with membranes (Mecozzi et al., 2014; Deatherage et al., 2020); an observation that is consistent with single-particle cryo-EM data indicating that dimers and tetramers were the prevalent retromer species in vitrified ice (Chen et al., 2019).

A very interesting aspect highlighting the role of the retromer in the pathophysiology of some misfolding diseases of the brain emerged from structural studies on the proteins forming this complex assembly, proteins which are defective in Alzheimer’s disease. In fact, depleting the retromer complex of the core’s central protein (called VPS35) is associated to the disruption of physiological recycling functions; therefore, the APP is not addressed to the Golgi as normal but to the lysosome, with an increase in the cleavage of APP and thus an enhanced formation of the Aβ40/42 amyloidogenic peptides feeding the growth of amyloid plaques. Small and Petsko (2015) and Small and Petsko (2020) went further presenting a set of VPS35 depletion–repletion studies in mice and even selecting small molecular weight pharmacological chaperones that have beneficial effects on the stability of the retromer*.*

### Intrinsically Disordered Proteins: Frustration and Disorder

As briefly recalled previously, Anfinsen’s experiments demonstrated the presence in proteins of a strong energetic bias toward the native conformation, resulting in spontaneous folding. One of the most elegant theories to explain folding implies the so-called principle of minimal frustration, which postulates proteins to display a minimal degree of frustration. In physical systems, frustration occurs when it is impossible to simultaneously minimize the free energy of all the possible interactions (Bryngelson et al., 1995; Onuchic et al., 1996). A critical corollary of such a principle implies that the amino acidic sequence of a polypeptide is optimal for its corresponding native structure. In line with Anfinsen’s observations, the free energy landscape appears funneled and strongly biased toward the native state. Recalling that proteins are principally optimized to function, the evolutionary constraint on function might be remarkably different from that of optimizing folding. Therefore, it is observed that proteins contain local frustration of non-optimized structure that often overlaps with their functional sites (Ferreiro et al., 2007; Ferreiro et al., 2011; Gianni et al., 2014). Local frustration is therefore a signature of the conflicting demands between folding and function.

**FIGURE 3:**
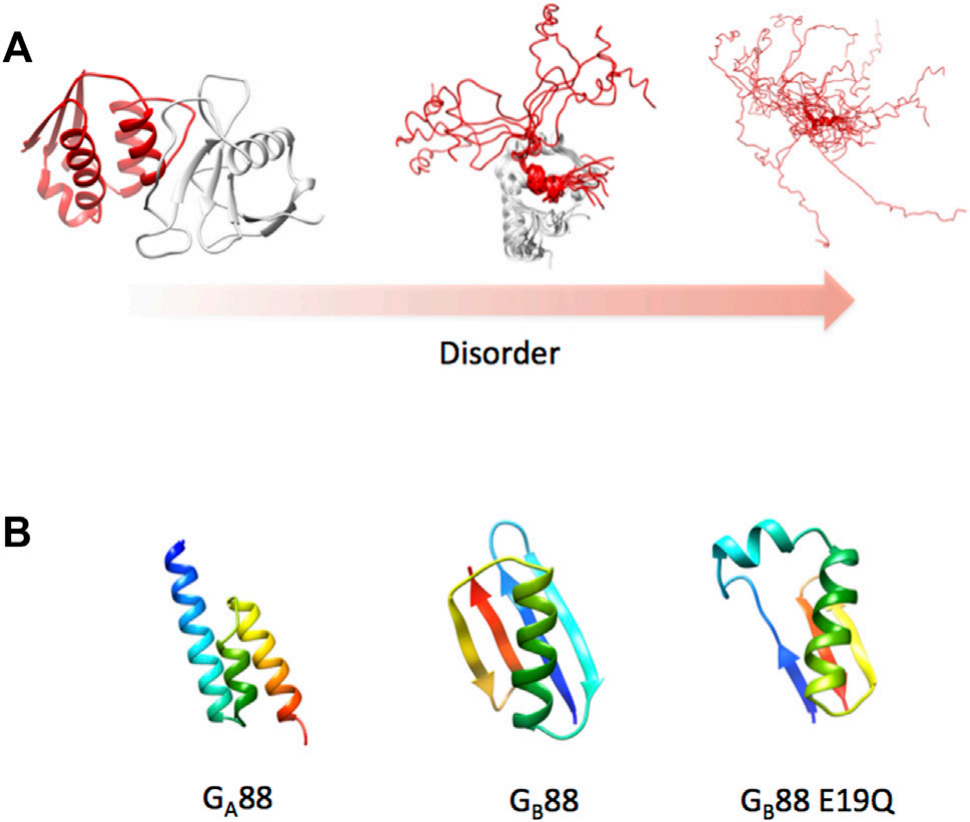
Protein structure and disorder. **(A)** As discussed in the text, the concept of a native state has been recently enriched by the identification of a disorder that characterizes several proteins. To exemplify this concept, we report three different cases characterized by a remarkable difference in the protein disorder. The pbd codes of the different cases are 1ay7, 2ly4, and 2fft (from left to right). **(B)** 3D structure of Gα88, Gβ88, and the variant E19Q of Gβ88 as predicted by the AlphaFold algorithm. Structures were obtained at the web server reported in Mirdinta et al. (2021).

As championed by Hans Frauenfelder (Austin et al., 1973; Frauenfelder et al., 1991; Frauenfelder et al., 2001), it may be assumed that all proteins possess some degree of frustration, sampling a continuum between the order (low frustration) and disorder (high frustration). Since frustration promotes the formation of multiple local minima in a free energy landscape, its increase would imply the population of different conformational sub-states resulting in more heterogeneous ensembles (Austin et al., 1973; Frauenfelder et al., 2001; Gianni et al., 2021). This leads to the emergence of a structural multiplicity which may be manifested in different ways such as (i) different alternative secondary structures; (ii) conformations of flexible linkers, dictating tertiary long distance interactions; or (iii) highly heterogeneous disordered domains populating different interconvertible conformations (Figure 3). Proteins possessing such very broad structural ensembles are often denoted as “intrinsically disordered proteins” (IDP). In this case, the native state is in fact a conformational ensemble of rapidly interconverting states, displaying very different properties compared to the relative order of a canonical globular protein.

The discovery that a considerable fraction of the human proteome contains a significant quota of dynamic disorder has substantially changed the outlook on our understanding of the linkage between the structure and function (Dunker et al., 2008; Uversky and Dunker 2010; Tantos et al., 2012; Uversky 2018). In fact, since IDPs are abundant and often very important in cell metabolism, a recurrent question in molecular bioscience is whether there is a hitherto overlooked potential value of protein disorder. More than one hypothesis has been put forward to unveil the significance of disorder. From a thermodynamic viewpoint, it has been suggested that the destabilization of a structured native state may result in reduction of affinity for the physiological ligand, without necessarily jeopardizing specificity (Spolar and Record 1994; Uversky 2018). The latter model represents to date one of the most controversial aspects (Gianni and Jemth 2019). From a kinetic viewpoint, it has been suggested that the IDPs populate an increased capture radius to recognize a partner (Shoemaker et al., 2000). In fact, a disordered protein could form with a physiological partner an extended high-energy complex that would be locked in place by the induced folding reaction. Importantly, it should be stressed that not all IDPs necessarily fold upon binding to their physiological partners, and a broad spectrum of disorders may still be found in bound complexes. Moreover, the presence of disordered stretches connecting folded domains might be crucial in regulating their transient interaction, thereby providing an allosteric platform to regulate function. Finally, more recently, IDPs have been associated with their capability to assemble in cell membrane-less organelles, thereby contributing to compartmentalization of the cellular environment.

### Predicting the Atomic Structure of the Native State

The general concept that emerged from Anfinsen’s experiment is that the amino acid sequence of a protein contains in itself all the information necessary to fold to the native state, in water. Thus, it was clear that the 3D structure of a protein could, in principle, be calculated from the amino acid sequence, provided the code relevant to morphogenesis was cracked. Over the last 50 years, many smart scientists have engaged in attempts to solve this terribly difficult problem, with fantastic but not definitive solutions. To evaluate progress and praise the best models for predicting the protein structure, an international competition called Critical Assessment of Protein Structure Prediction (CASP) has been organized every 2 years since 1994 by Moult et al. (1995). Our friend and colleague, the late Anna Tramontano, Professor of Biochemistry in Rome, represented a critical personality engaged in running several CASP meetings until 2016, when she sadly passed away.

In 2020, at the 14th CASP event, it was recognized that a Google algorithm called AlphaFold based on artificial intelligence allowed to calculate the 3D structure of a globular protein in water to an unprecedented accuracy that rivaled the best experimental structures (Kryshtafovych et al., 2021). The success achieved by AlphaFold seems to result from the remarkable improvements of the artificial intelligence methodologies in general and their applications to biological systems (Thorn 2022). At the same CASP meeting, David Baker reported exciting results comparable to AlphaFold obtained with an extension of his algorithm called the Rosetta fold (Humphreys et al., 2021). Remarkably, these innovative techniques appear to return in few minute predictions whose reliability is comparable to the experimental uncertainties arising in crystallographic experiments.

As an example for the predicting power of AlphaFold, we ran a structural prediction on two particularly difficult proteins, named Gα88 and Gβ88, sharing an 88% sequence identity but displaying completely different 3D structures (Alexander et al., 2007). Because of the exceptionally high-sequence identity of these two structurally different proteins, running this test would appear particularly challenging for the algorithm. Remarkably, AlphaFold correctly predicted the two alternative structures (as shown in Figure 3) despite the fact that the two proteins differed by only 7 out of 56 amino acid residues. In the course of our study, we designed a single-point mutation of Gβ88, where Glu19 was mutated to Gln, and observed by CD spectroscopy an increase in the propensity of Gβ88 to partially populate a helical fold (Gianni et al., 2018). To our delight, subjecting the mutated sequence to AlphaFold, the program correctly predicted such a structural heterogeneity returning different models, which include among others a conformation that appears hybrid between Gα88 and Gβ88 (Figure 3B). The extraordinary performance of AlphaFold will be extremely valuable to investigate the role of IDPs, considering that the program is exceptionally user-friendly and freely available to the scientific community (Mirdinta et al., 2021).

## Concluding Remarks

Since the pioneering work of Anfinsen and his co-workers (1961), our understanding of the *native* state of a protein has been enormously enriched. In fact, the initial assumption that protein function is fully compatible with the structural architecture of a relatively static macromolecule has been challenged long ago by introducing a more complex picture, taking into account both the dynamics of the polypeptide and its tendency to aggregate. Importantly, the large repertoire of folds of native globular proteins, each dictated by a genetically determined sequence, underlies the Darwinian selection process driven by functional evolutionary pressure; a mechanism that notably has not emerged for the amyloid fold as far as we know. In this context, it is accepted that dynamics, frustration, and local instability are often crucial components of protein function, at variance with the monotonous structure, the minimal frustration, and the pronounced stability of an amyloid. Since the latter state is generally very stable and resistant to heat or proteolytic attack, it may be said that a protein molecule captured into an amyloid is immortalized, this state being incompatible with the life of the cell. To close, it may be appropriate to quote the French poet Paul Claudel: *“L’ordre est le plaisir de la raison; mais le désordre est le délice de l’imagination.”*
